# Single Cell Analysis of Transcriptional Activation Dynamics

**DOI:** 10.1371/journal.pone.0010272

**Published:** 2010-04-21

**Authors:** Ilona U. Rafalska-Metcalf, Sara Lawrence Powers, Lucy M. Joo, Gary LeRoy, Susan M. Janicki

**Affiliations:** 1 Gene Expression and Regulation Program, The Wistar Institute, Philadelphia, Pennsylvania, United States of America; 2 Department of Molecular Biology, Princeton University, Princeton, New Jersey, United States of America; Institut Curie, France

## Abstract

**Background:**

Gene activation is thought to occur through a series of temporally defined regulatory steps. However, this process has not been completely evaluated in single living mammalian cells.

**Methodology/Principal Findings:**

To investigate the timing and coordination of gene activation events, we tracked the recruitment of GCN5 (histone acetyltransferase), RNA polymerase II, Brd2 and Brd4 (acetyl-lysine binding proteins), in relation to a VP16-transcriptional activator, to a transcription site that can be visualized in single living cells. All accumulated rapidly with the VP16 activator as did the transcribed RNA. RNA was also detected at significantly more transcription sites in cells expressing the VP16-activator compared to a p53-activator. After α-amanitin pre-treatment, the VP16-activator, GCN5, and Brd2 are still recruited to the transcription site but the chromatin does not decondense.

**Conclusions/Significance:**

This study demonstrates that a strong activator can rapidly overcome the condensed chromatin structure of an inactive transcription site and supercede the expected requirement for regulatory events to proceed in a temporally defined order. Additionally, activator strength determines the number of cells in which transcription is induced as well as the extent of chromatin decondensation. As chromatin decondensation is significantly reduced after α-amanitin pre-treatment, despite the recruitment of transcriptional activation factors, this provides further evidence that transcription drives large-scale chromatin decondensation.

## Introduction

During transcriptional activation, gene specific activators bind to their response elements and recruit the regulatory factors needed to initiate efficient RNA synthesis. The basic steps in this process include chromatin decondensation, pre-initiation complex assembly, and RNA polymerase II (RNA pol II) elongation through the gene [1], [2]. Transcriptional output is also a product of activator strength and likely depends on the regulatory factors each recruits as well as their recruitment timing [3]. Increasing the number of activator binding sites can also have a synergistic effect on activation and has been shown to eliminate the need for specific regulatory steps such as histone acetylation [4].

However, in all cases, transcriptional activators must overcome the significant impediment that chromatin structure imposes on transcription. In order for the pre-initiation complex to form and RNA synthesis to proceed, the DNA duplex must be opened, which requires decondensation of high-order chromatin structure and nucleosome disassembly [5], [6]. Interestingly, many elongation factors are histone chaperones highlighting the importance of nucleosomal dynamics in this process. For example, FACT (facilitates chromatin transcription), composed of the HMG-1-like protein, SSRP1 and p140/Spt16, transiently binds and removes H2A/H2B dimers from nucleosomes [7]. Additionally, nucleolin is required for RNA pol I elongation [8]; Asf1 mediates nucleosome disassembly at the PHO5 and PHO8 promoters [9]; and Brd proteins, discussed more extensively below, function as chaperones and facilitate elongation on acetylated chromatin templates in a defined transcription system [10].

Enzymes that add and remove protein post-translational modifications (PTMs) are also important transcriptional regulators because they create and eliminate regulatory factor binding sites [11]. The histone proteins – particularly their N-terminal tails – are important targets, but other factors, including transcription factors, are also modified [12], [13]. Specific histone PTMs are associated with silent and active chromatin. Histone lysine acetylation is enriched at active chromatin and the bromodomain (BD) is the protein module that binds acetylated lysines. BDs are found in histone acetyltransferases (HATs), including GCN5, PCAF and p300, chromatin remodeling factors, general transcription factors and elongation factors [11], [14], [15].

Members of the BET (bromodomains and extraterminal) protein family, found in yeast and animals, contain tandem BDs and are of great interest because of their roles in regulating early events in transcriptional activation [16]. In mammals, Brd2, Brd3 and Brd4 are widely expressed and Brdt is testes specific. Brd proteins also regulate chromatin organization and epigenetic inheritance and are required for development – functions that all require their BDs and/or their ability to bind acetylated lysines. Acetyl-lysine binding is also required for Brd2 and Brd4 to both interact with and mediate the attachment of episomal viral genomes to mitotic chromosomes [17], [18], [19], [20], [21], [22]. In yeast, Bdf1 prevents heterochromatin spreading by competing with Sir2 for acetylated H4 at euchromatin-heterochromatin boundaries [23], [24]. Brd4 +/− cells are hypo-acetylated at H3 K14 and H4 K12 suggesting that Brd4 preserves both its own binding sites as well as global acetylation [25]. When over-expressed, Brd2 increases histone H4 acetylation and gene activation [17], [26], [27].

Most studies of transcriptional activation indicate that regulatory events occur in a temporally defined order [28], [29], [30]. However, as they were mostly done utilizing molecular genetic and biochemical methods, in which effects are averaged in cell populations, it remains to be seen how they are dynamically regulated in single cells. Transcriptional activators bind to specific sequence elements in genes and initiate the recruitment of the molecular machines needed to decondense chromatin and activate transcription. Unlike activators though, most of these recruitment events are mediated by a complex array of dynamic protein-protein and protein-PTM interactions [31]. Although many have been extensively characterized using biochemistry, it is not well understood how they are coordinated at transcription sites in relation to changes in chromatin organization and the initiation of RNA synthesis.

Advances in imaging technology and the development of methods that allow gene expression to be tracked in real time in single cells now make it possible to address these types of questions [32], [33]. In *Drosophila* salivary gland tissue, polytene chromosomes provide the signal intensity needed to identify individual genes by chromosome banding and have been used to study gene activation in a natural chromatin environment [32]. The RNA pol II subunit, Rpb3, and heat shock factor (HSF), the hsp70 transcriptional activator, rapidly accumulate at the native *hsp70* gene loci after induction [34], [35]. As specific loci cannot be similarly analyzed in mammalian interphase cells, other systems have been developed in order to do this.

The multi-copy insertion of transcriptional reporter constructs into single genomic sites in mammalian cells provides the signal intensity needed to investigate transcriptional dynamics at a defined site. A cell line with a multi-copy array (∼200 copies) of the mouse mammary tumor virus/Harvey viral ras (MMTV/v-Ha-ras) reporter has been used to show that the glucocorticoid receptor (GR) associates dynamically with its binding sites at a natural promoter [36]. However, as the LTR only has 4 GR binding sites (∼800–1200 total in array) [37], it is difficult to utilize this interaction to track factor recruitment at the earliest stages of activation. Additionally, the mRNA can only be detected using RNA FISH making it impossible to study how RNA synthesis is dynamically coordinated with other regulatory events [38].

To address these issues, arrays of bacterial and bacteriophage sequence element repeats have been introduced into inducible transgenes. Fusion of their requisite binding proteins to auto-fluorescent proteins allows these DNA and RNA elements to be used to directly visualize gene activity in single mammalian cells [33]. Multi-copy integration of these transgenes provides the signal intensity needed for factor accumulation, RNA synthesis and chromatin decondensation to be clearly monitored throughout the course of activation. Additionally, the inactive transcription site can be visualized in intact cells, changes in chromatin architecture can be correlated to RNA synthesis, and factor recruitment and regulatory pathway coordination can be investigated.

In this study, we use such a system [39] to examine the recruitment of transcriptional regulatory factors to a heterochromatic transcription site during activation. GCN5, RNA pol II and Brd4 are all rapidly recruited with a VP16-transcriptional activator. In contrast, Brd2, which requires its BDs for recruitment, lags behind the activator by ∼2 minutes. Interestingly, RNA accumulates at the site simultaneously with the VP16-activator indicating that a strong activator can rapidly overcome a condensed chromatin environment and bypass the need for the expected ordered recruitment of regulatory factors before transcription can commence. As RNA accumulates at the transcription site in significantly more cells expressing the VP16-activator compared to a p53-activator, this demonstrates the importance of activator strength in this process. Additionally, transcriptional activation factors are still recruited to the transcription site in α-amanitin pre-treated cells. However, chromatin decondensation is significantly decreased compared to control cells providing further evidence for the essential role of transcription in driving large-scale changes in chromatin architecture [40], [41].

## Results

### Description of the experimental system

**Figure 1:**
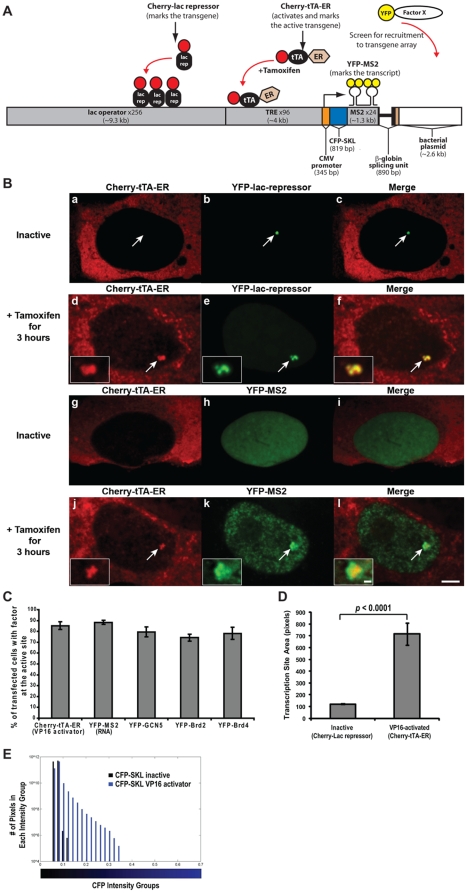
Description of the live-cell gene expression system. (**A**) Schematic diagram of the inducible transgene drawn to scale. The interaction of Cherry-lac repressor with the lac operator repeats allows the transgene to be visualized in both the inactive and active states. Cherry-tTA-ER (Cherry-tetracycline transcriptional activator-estrogen receptor) binds to the tetracycline response element (TRE) repeats in the presence of tamoxifen and activates transcription from the CMV minimal promoter. The transcribed RNA includes CFP fused to a peroxisomal targeting signal (CFP-SKL), 24 MS2 translational operators (MS2 repeats) and a splicing unit and the 3′ UTR from the rabbit β-globin gene. The RNA is visualized through the interaction of YFP-MS2 coat protein dimers with the MS2 translational operators (stem loop structures). YFP-tagged proteins co-expressed with either Cherry-lac repressor or Cherry-tTA-ER can be evaluated for recruitment to the inactive and active transcription site, respectively. Repeat elements in the transgene are shaded gray; the length of each is shown in parentheses. (**B**) Localization of the proteins that mark the inactive and active transcription site. Cherry-tTA-ER is sequestered in the cytoplasm (panels **a** and **g**) until the addition of tamoxifen induces its entry into the nucleus where it both activates transcription and marks the active site (panels **d** and **j**). YFP-lac repressor constitutively binds to the lac operator repeats, and therefore, marks both the inactive (panel **b**) and active (panel **e**) transcription site. YFP-MS2 is diffusely distributed throughout the nucleus before activation (panel **h**). It accumulates at the transcription site and becomes particulate throughout the nucleus after activation (panel **k**). Scale bar represents 5 µm. Scale bar in the enlarged inset represents 1 µm. (**C**) Graph represents the percentage of transfected cells with accumulation of the regulatory factors at the transcription site 3 hr after activation. 100 cells were analyzed from 3 independent experiments. Average values and SEM (in the form of error bars) are presented in the graphs. (**D**) Measurement of the pixel area of the inactive transcription site (marked by Cherry-lac repressor) and VP16-activated transcription sites (marked by Cherry-tTA-ER) 3 hrs after activation. Values represent the averages of 10 cells. SEM (in the form of error bars) and a *p* value is presented in the graph. (**E**) Frequency histogram showing the distribution of the blue pixel intensity levels (blue bars) as a measure of the CFP-SKL protein in inactive and activated cells. Black bars represent the background signal. The x-axis is the average fluorescence pixel intensity in each bin on a scale from 0 to 1 and divided into bin sizes of 0.02; the y-axis is the number of pixels in each bin, on a logarithmic scale. The bar beneath the histogram shows the intensity range. 10 independent cells were analyzed.

In order to study the dynamics of gene expression in living cells, we previously developed a cell line (2-6-3) in which we can simultaneously visualize a transcription site and the RNA and protein produced from it in real time in single cells [39]. It is derived from the human osteosarcoma cell line, U2OS, and contains a stable multi-copy chromatinized array of an inducible transgene (∼200 copies integrated into a euchromatic region of chromosome 1). The transgene contains lac operator repeats (256 copies), which allow it to be visualized in both the inactive and active states when lac repressor fused to an auto-fluorescent protein, Cherry [42] or YFP, is expressed (Figs. 1A and1B, panels b and e).

Transcription is induced using the Tet-On/Tet-Off system. When tetracycline-regulated activators bind to the tetracycline response elements (TREs; 96 copies), transcription is driven from the CMV minimal promoter (Fig. 1A). In previous studies, we used the reverse tet transcriptional activator (rtTA; reverse tet repressor fused to VP16), which binds to the TREs in the presence of doxycycline. However, rtTA localizes throughout the nucleus and cytoplasm and is prone to transcriptional leakiness. In order to achieve tighter transcriptional regulation and to use the activator/DNA interaction to visualize the transcription site, we fused the tetracycline transcriptional activator (tTA; tet repressor fused to VP16), to Cherry and the hormone binding domain of the estrogen receptor (ER) [43] to create the tamoxifen-inducible Cherry-tTA-ER (Fig. 1A). Before activation, Cherry-tTA-ER is sequestered in the cytoplasm and cannot be detected at the transcription site, marked by YFP-lac repressor confirming that the system is not leaky (Fig. 1B, panels a–c; Table S1). After the addition of tamoxifen, a population of Cherry-tTA-ER enters the nucleus, accumulates at the transcription site, and co-localizes with YFP-lac repressor in 85.2±3.6% of transfected cells (Fig. 1B, panels d and j; Fig.1C; Table S1). The majority of Cherry-tTA-ER, however, remains in the cytoplasm after activation.

In the inactive state, the transcription site is highly condensed and, as previously reported, is enriched for the heterochromatic histone modification, H3 tri-meK9, and proteins (HP1s) [39]. To determine the effect of transcriptional activation on the condensation state of the transcription site chromatin, we measured and compared the pixel area of the inactive (marked by Cherry-lac repressor) and active (marked by Cherry-tTA-ER) sites (Fig. 1D). The active site occupies ∼6 times the area of the inactive site demonstrating that the chromatin significantly decondenses during activation (Fig. 1D).

The transcribed RNA encodes cyan fluorescent protein (CFP) fused to a peroxisomal targeting signal (SKL), 24 repeats of the MS2 bacteriophage translational operator and a splicing cassette and the 3′ UTR from the rabbit β-globin gene. This RNA can be visualized by expressing the YFP-MS2 binding protein, which binds to the stem loop structure of the MS2 translational operator as a dimer (Fig. 1A). 3 hours after activation, YFP-MS2 accumulates at the transcription site in 88.3±1.8% of cells (Fig. 1B, panel k; Fig. 1C; Table S1). There are also a greater number of pixels with high CFP signal intensity in cells activated for 3 hours compared to inactive cells indicating that CFP-SKL is highly expressed and confirming that the mRNA is processed, exported and translated [39] (Fig. 1E).

**Figure 2:**
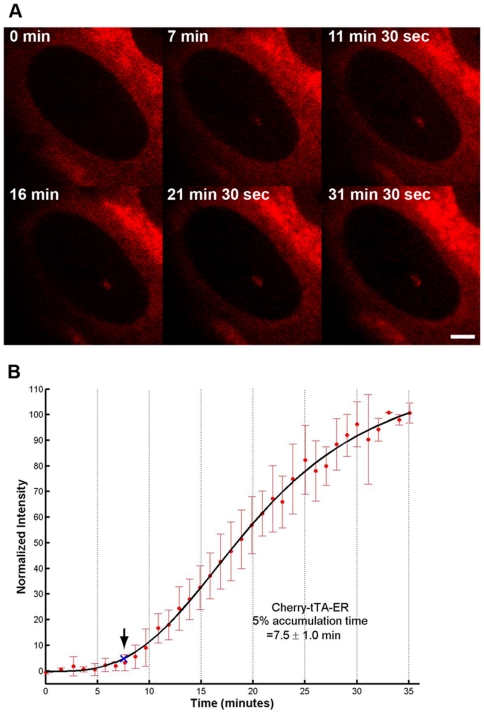
Real-time analysis of the recruitment of the VP16-transcriptional activator. (**A**) Still images from a time series of 2-6-3 cells expressing Cherry-tTA-ER during activation. Selected images taken from Movie S1 show the accumulation of the VP16 activator at the transcription site. Scale bar is equal to 5 µm. (**B**) Quantification of Cherry-tTA-ER recruitment to the transcription site during activation. Tamoxifen was added to the media immediately after the first time point (∼0 min). Images were collected every 1 min for ∼40 min. Measured intensities were normalized to the high and low plateau values and fitted to a logistic fit (solid black line). The initial accumulation time (blue X and arrow) is the point when the fitted curve reaches 5% of the total accumulation. The graph is the average of 6 cells imaged from 4 different coverslips on 3 different days. Error bars represent the standard deviation.

To determine the timing of transcriptional activation, we monitored the recruitment of Cherry-tTA-ER to the transcription site using time-lapse imaging (Fig. 2A; Movie S1). For this analysis, cells expressing Cherry-tTA-ER at equivalent intensity levels were selected for imaging. The levels of this factor at the transcription site were determined and fit to a logistical model and a good agreement was seen between the data and the fit curve. Using this model, the start time of accumulation was defined as the point when the signal intensity at the transcription site reached 5% of the total value between the normalized initial baseline (0% signal) and the final total accumulation (100% signal). At this threshold, the signal intensities are above background levels but not yet in the regions of rapid accumulation where varying accumulation rates would distort initial time measurements. Under these parameters, Cherry-tTA-ER is recruited 7.5±1.0 min after the addition of tamoxifen (Fig. 2B; Table S2).

### Histone acetyltransferases are strongly and rapidly recruited to the transcription site

**Figure 3:**
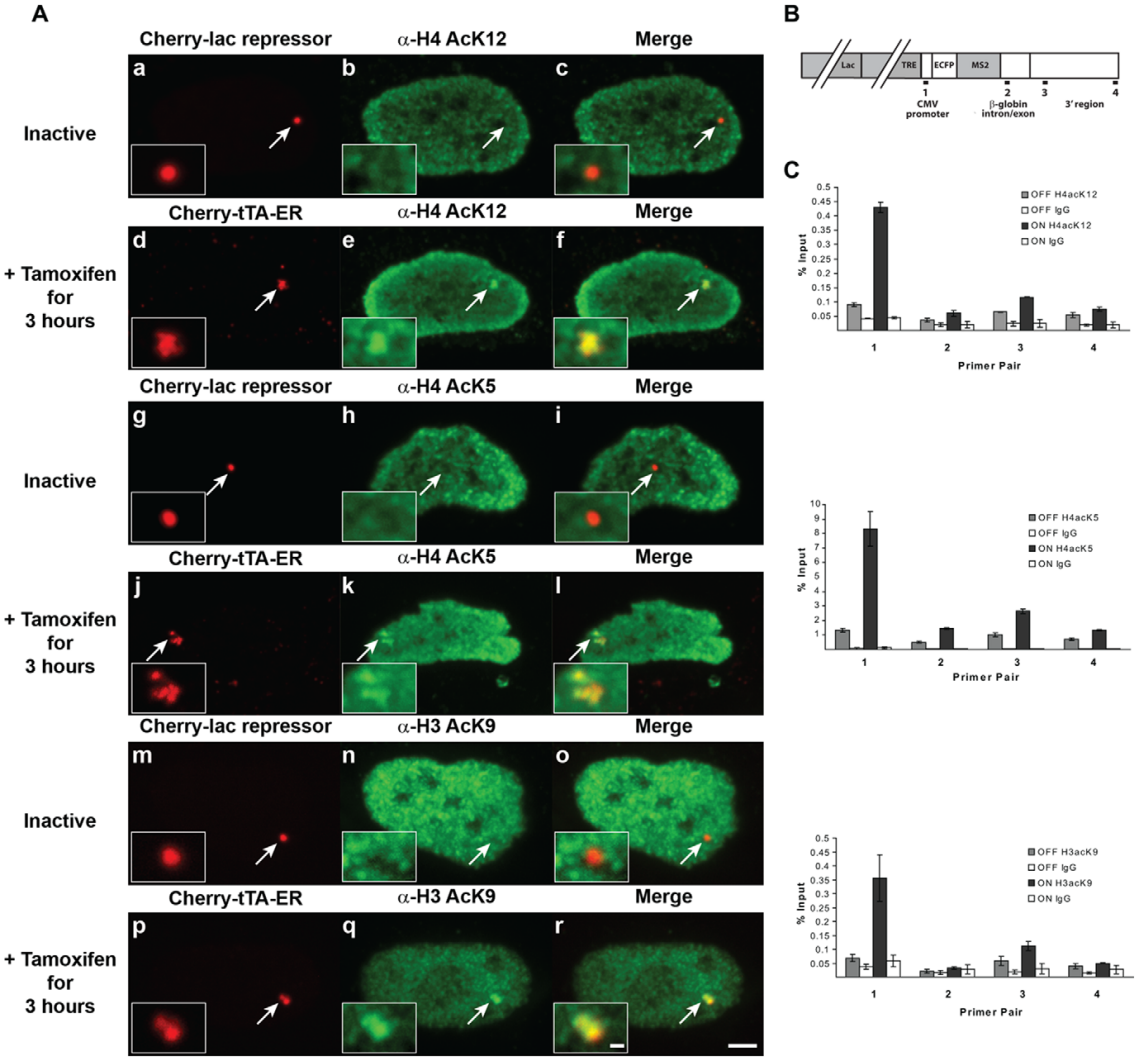
Immunofluorescence and ChIP analyses of histone acetyl-lysine modifications at the transcription site. (**A**) Histone H4 acetyl-K12, histone H4 acetyl-K5 and histone H3 acetyl-K9 levels at the transcription site were visualized by immunostaining. H4 acetyl-K12 (panels **a**–**c**), H4 acetyl-K5 (panels **g**–**i**) and H3 acetyl-K9 (panels **m**–**o**) are not enriched at the inactive site, marked by Cherry-lac repressor. H4 acetyl-K12 (panels **d**–**f**), H4 acetyl-K5 (panels **j**–**l**) and H3 acetyl-K9 (panels **p**–**r**) are enriched at the active site, marked by Cherry-tTA-ER. Transcription was activated for 3 hr by the addition of tamoxifen. Scale bar is equal to 5 µm. Scale bar in enlarged inset is equal to 1 µm. (**B**) Schematic representation of the inducible transgene shows the location of primers and probes used for real-time PCR in the ChIP assay. (**C**) The presence of the histone acetyl-lysine modifications along the transgene in transcriptionally inactive and active cells was analyzed using ChIP. Results were obtained from 3 independent experiments. Average values and SEM (in the form of error bars) are presented in the graphs.

To evaluate the changes that occur to the transcription site chromatin during activation, we immunostained inactive and transcriptionally activated cells using antibodies against histone H4 acetyl-K5, histone H4 acetyl-K12, and histone H3 acetyl-K9, which are binding sites for the acetyl-lysine binding, Brd2 and Brd4 proteins [10], [17], [44], [45], [46]. Figure 3A and Table S1 show that they are only enriched at active transcription sites. These antibodies were also used for chromatin immunoprecipitation (ChIP) to determine the sequence elements at which they are enriched (primer pairs Fig. 3B). All of the acetyl-lysine modifications were strongly and specifically enriched at the promoter after activation (Fig. 3C).

**Figure 4:**
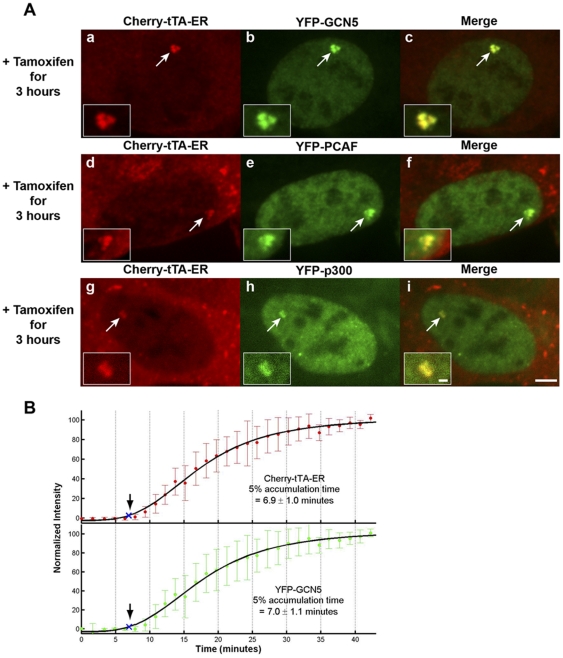
Analysis of histone acetyltransferases dynamics during activation. (**A**) YFP-GCN5 (panels **a**–**c**), YFP-PCAF (panels **d**–**f**), and YFP-p300 (panels **g**–**i**) are strongly recruited to the active transcription site, marked by Cherry-tTA-ER, 3 hr post induction. Transcription was induced by the addition of tamoxifen. Scale bar represents 5 µm. Scale bar in the enlarged inset represents 1 µm. (**B**) Quantification of Cherry-tTA-ER (top panel, solid red circles) and YFP-GCN5 (lower panel, solid green circles) accumulation at the transcription site during activation. Tamoxifen, was added to the media immediately after the first time point (∼0 min). Images were collected every 1.5 min for ∼40 min. Measured intensities were normalized to the high and low plateau values and fitted to a logistic fit (solid black line). The initial accumulation time (blue Xs and arrows) is the point when the fitted curve reaches 5% of the total accumulation. The graphs are the average of 7 cells imaged from 4 different coverslips on 4 different days. Error bars represent the standard deviation.

Since we detected strong accumulation of histone lysine acetylation at the active site (Fig. 3), we also wanted to identify the histone acetyltransferases (HATs) recruited to it. YFP-tagged GCN5, PCAF and p300 were not enriched at the inactive site, marked by Cherry-lac repressor (Fig. S1A; Table S1). However, all were strongly recruited and co-localized with Cherry-tTA-ER upon activation (Fig. 4A; Table S1).

YFP-GCN5 was enriched at the transcription site in 79.5±4.5% of activator-expressing cells (Fig. 1C and S1B). To determine its recruitment timing in relation to Cherry-tTA-ER, we collected time-lapse images of cells expressing these factors during activation. Cherry-tTA-ER and GCN5 reached the 5% accumulation threshold 6.9±1.0 and 7.0±1.1 min after the addition of tamoxifen, respectively (Fig. 4B; Movie S2; Table S2). As described above, the 5% threshold was set as the start point of accumulation. To validate this parameter value, the times at the 2.5 and 10% thresholds were also examined and shown to exhibit identical trends (Table S2). Consistency across a range of values for this parameter is within the error of the logistic fit and the limits of our time resolution and supports the conclusion that GCN5 is recruited with the activator.

### RNA synthesis begins rapidly upon transcriptional activator recruitment

**Figure 5:**
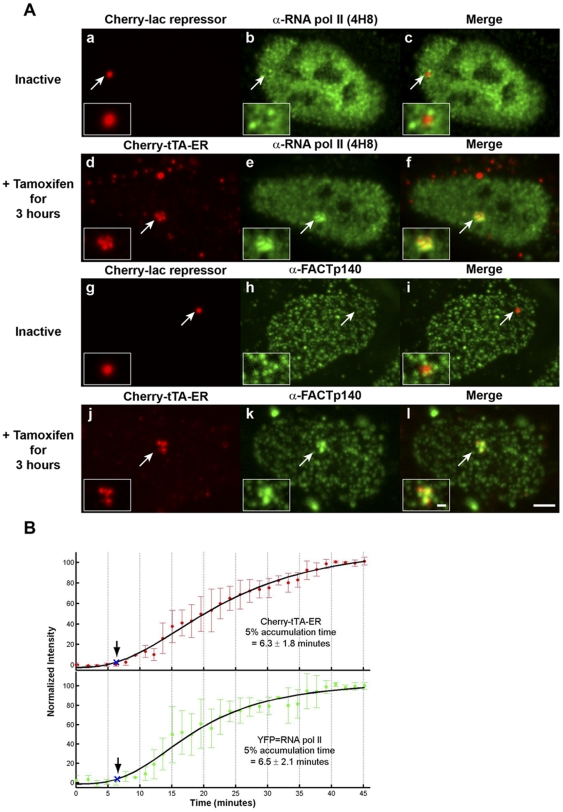
Analysis of RNA pol II and FACTp140 at the transcription site. (**A**) RNA pol II (panels **a**–**c**) and the FACT subunit p140/Spt16 (panels **g**–**i**) surround but do not co-localize with the inactive transcription site, marked by Cherry-lac repressor. RNA pol II and p140/Spt16 are strongly enriched and co-localize with the active transcription site, marked by Cherry-tTA-ER (panels **d**–**f** and **j**–**l**). Transcription was induced by the addition of tamoxifen and cells were fixed and stained 3 hr later. Scale bar represents 5 µm. Scale bar in enlarged inset represents 1 µm. (**B**) Quantification of Cherry-tTA-ER (top panel, solid red circles) and YFP-RNA pol II (lower panel, solid green circles) accumulations at the transcription site during activation. Tamoxifen, was added to the media immediately after the first time point (∼0 min). Images were collected every 1.5 min for ∼40 min. Due to the low intensity of the YFP-RNA pol II signal, a gamma correction of 2 was applied to each image in both channels to distinguish factor accumulation from background at the early time points. Measured intensities were normalized to the high and low plateau values and fitted to a logistic fit (solid black line). The initial accumulation time (blue Xs and arrows) is the point when the fitted curve reaches 5% of the total accumulation. The set of graphs are the average of 4 cells imaged from 4 different coverslips on 3 different days. Error bars represent the standard deviation.

We next evaluated the localization of RNA pol II and the FACT subunit, p140/Spt16, at the transcription site. Using immunofluorescence (IF), we found that neither co-localized with the inactive site although accumulations were detected around its periphery (Fig. 5A, panels a–c and g–i; Table S1). Similar results were obtained in YFP-RNA pol II expressing cells (Fig. S2, panels a–d). However, both RNA pol II and p140/Spt16 were strongly recruited to the active transcription site marked by Cherry-tTA-ER (Fig. 5A, panels d–f and j–l; Fig. S2, panels e–h; Table S1).

In order to determine the timing of RNA pol II recruitment, we collected time-lapse images of cells co-expressing YFP-RNA pol II and Cherry-tTA-ER during activation. Cherry-tTA-ER accumulated 6.3±1.8 min and YFP-RNA-pol II accumulated 6.5±2.1 min after tamoxifen addition, indicating that RNA pol II is rapidly recruited upon activator binding (Fig. 5B; Movie S3; Table S2).

**Figure 6:**
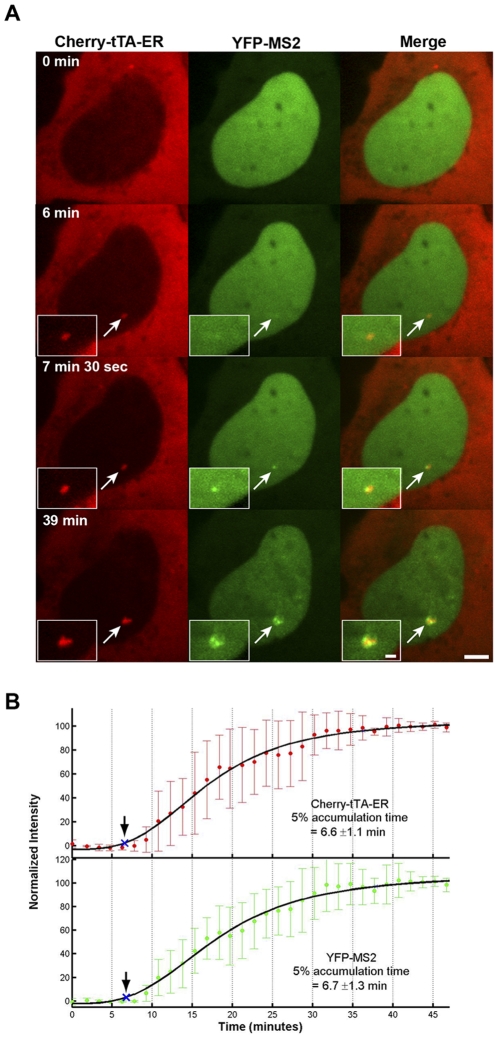
Real-time analysis of mRNA synthesis. (**A**) Still images from a time series of 2-6-3 cells expressing Cherry-tTA-ER and YFP-MS2. Selected images from Movie S4 show their accumulation at the transcription site during activation. Scale bar represents 5 µm. Scale bar in enlarged inset represents 1 µm. (**B**) Quantification of Cherry-tTA-ER (top panel, solid red circles) and YFP-MS2 (lower panel, solid green circles) accumulations at the transcription site during activation. Tamoxifen was added to the media immediately after the first time point (∼0 min). Images were collected every 1.5 min for ∼40 min. Measured intensities were normalized to the high and low plateau values and fitted to a logistic fit (solid black line). The initial accumulation (blue Xs and arrows) is when the fitted curve reaches 5% of the total accumulation. The set of graphs represents the average of 8 cells imaged from 6 different coverslips on 4 different days. Error bars represent the standard deviation.

In order to determine when mRNA synthesis begins, we measured YFP-MS2 levels (6.7±1.3 min) at the transcription site in relation to Cherry-tTA-ER (6.6±1.1 min) (Fig. 6; Movie S4). As the timing was almost identical (Fig. 6B), this indicates that transcription is rapidly induced upon activator binding.

### Analysis of Brd2 and Brd4 recruitment during activation

**Figure 7:**
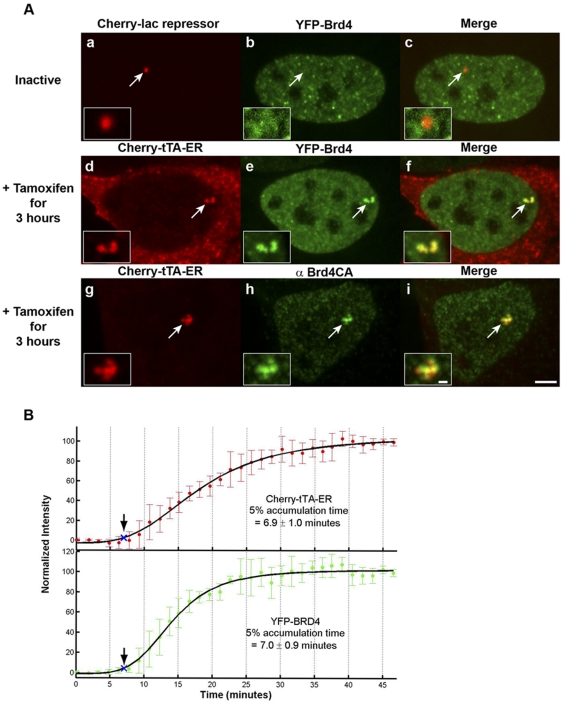
Analysis of Brd4 recruitment to the transcription site. (**A**) YFP-Brd4 is not enriched at the inactive transcription site marked by Cherry-lac repressor (panels **a**–**c**). YFP-Brd4 and endogenous Brd4, immunostained with antibody Brd4CA, are strongly recruited to the active site marked by Cherry-tTA-ER (panels **d**–**f** and **g**–**i**, respectively). Transcription was induced for 3 hr by the addition of tamoxifen. Scale bar represents 5 µm. Scale bar in enlarged inset represents 1 µm. (**B**) Quantification of Cherry-tTA-ER (top panel, solid red circles) and YFP-Brd4 (lower panel, solid green circles) accumulations at the transcription site during activation. Tamoxifen was added to the media immediately after the first time point (∼0 min). Images were collected every 1.5 min for ∼40 min. Measured intensities were normalized to the high and low plateau values and fitted to a logistic fit (solid black line). The initial accumulation (blue Xs and arrows) is when the fitted curve reaches 5% of the total accumulation. The set of graphs represents the average of 6 cells imaged from 4 different coverslips on 4 different days. Error bars represent the standard deviation.

**Figure 8:**
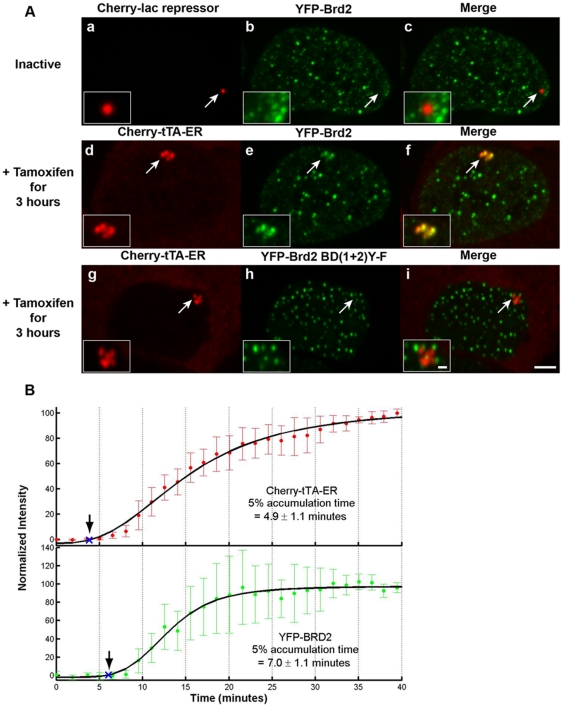
Analysis of Brd2 recruitment to the transcription site. (**A**) YFP-Brd2 is not enriched at the inactive transcription site marked by Cherry-lac repressor (panels **a**–**c**). YFP-Brd2 is strongly recruited to the active site marked by Cherry-tTA-ER (panels **d**–**f**). YFP-Brd2 BD(1+2) Y→F in which tyrosine (Y)113 and Y136 are mutated to phenylalanines (F) is not recruited to the active transcription site (panels **g**–**i**). Transcription was induced for 3 hr by the addition of tamoxifen. Scale bar represents 5 µm. Scale bar in enlarged inset represents 1 µm. (**B**) Quantification of Cherry-tTA-ER (top panel, solid red circles) and YFP-Brd2 (lower panel, solid green circles) accumulations at the transcription site during activation. Tamoxifen was added to the media immediately after the first time point (∼0 min). Images were collected every 1.5 min for ∼40 min. Measured intensities were normalized to the high and low plateau values and fitted to a logistic fit (solid black line). The initial accumulation time (blue Xs and arrows) is the point when the fitted curve reaches 5% of the total accumulation. The set of graphs represents the average of 5 cells imaged from 3 different coverslips on 3 different days. Error bars represent the standard deviation.

As the active transcription site is enriched with histone lysine acetylation and GCN5 rapidly accumulates upon activation, we also wanted to evaluate the recruitment of acetyl-lysine binding factors with roles in regulating transcriptional activation. Brd4 (Fig. 7 and S1B) and Brd2 (Fig. 8 and S1B) are double BD proteins that interact with histone H4 acetylated at K5 and K12 – PTMs enriched at the active site (Fig. 3) [10], [17], [44], [45], [46]. Neither was enriched at the inactive site (Fig. 7A, panels a–c and Fig. 8A, panels a–c; Table S1), but both were strongly recruited upon activation in 74.3±3.3% and 78.2±5.5% of transfected cells, respectively (Fig. 7A, panels d–f and Fig 8A, panels d–f; Fig. 1C; Table S1). Immunofluorescence (IF) staining with an antibody against Brd4 showed that the endogenous factor accumulates at the active site in a pattern similar to YFP-Brd4 (Fig. 7A, panels g–i; Table S1).

We next evaluated the recruitment timing of Brd4 and Brd2 in relation to Cherry-tTA-ER. Brd4 accumulated rapidly with the activator (Fig. 7B; Movie S5; Table S2) but, interestingly, Brd2 lagged behind by ∼2 minutes (Fig. 8B; Movie S6; Table S2). To investigate the role of acetyl-lysine binding on Brd2 recruitment, we evaluated a construct with mutations that prevent this interaction. Tyrosine (Y) 113 in BD1 and Y386 in BD2 in Brd2 mediate the interaction with acetylated lysines; Y to phenylalanine (F) conversions inhibit it [10], [17]. YFP-Brd2 BD(1+2) Y→F does not accumulate at the active site indicating that this interaction is mediated by acetyl-lysine binding (Fig. 8A, panels g–i; Table S1). Therefore, the time difference detected between activator and Brd2 recruitment provides a measure of the time required for the site to become hyper-acetylated.

Interestingly, Brd2 decreased the timing of activator recruitment by ∼2 min (Table S2). This is consistent with reports that Brd2 over expression enhances transcriptional activation and global histone acetylation [17], [26], [27]. Perhaps it makes the TRE binding sites more accessible to the activator. Interestingly, YFP-Brd4 did not have a similar effect on activator accumulation (Table S2) although its expression stimulates transcription of some cellular genes [44].

### Activator Strength Regulates the Degree of Activation

As RNA levels increase simultaneously with Cherry-tTA-ER (Fig. 6B), and Brd2 accumulation is delayed compared to the activator (Fig 8B), this suggests that the VP16 activator can rapidly overcome the heterochromatic repression at the site and initiate transcription before the chromatin is hyper-acetylated. However, as there are 96 activator-binding sites upstream of the CMV promoter (Fig. 1A), it is also possible that the rapid and strong activation is due to the number of VP16 molecules recruited.

**Figure 9:**
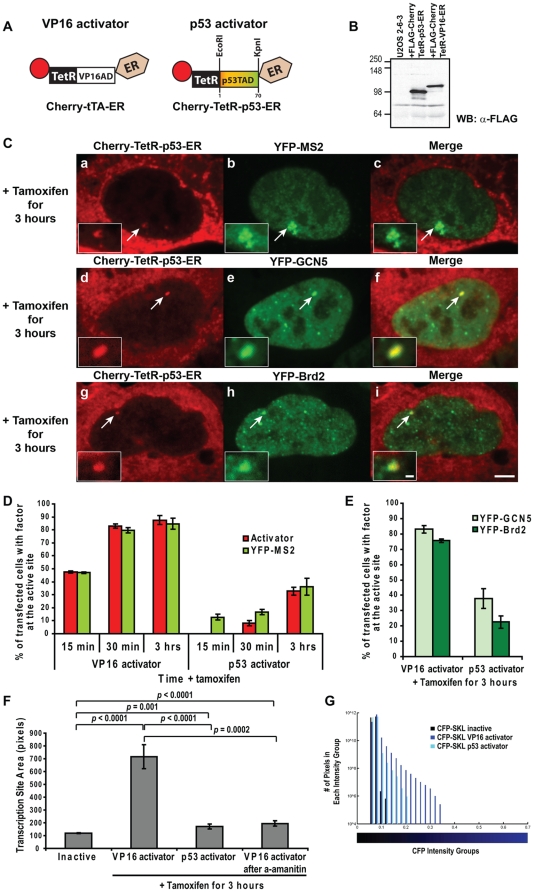
Single cell analysis of transcriptional activation induced by VP16 and p53 activators. (**A**) Schematic representation of the VP16 and p53 activators. (**B**) Western blot analysis of Flag-tagged versions of the p53 and VP16 activators showing that they are expressed at comparable levels in 2-6-3 cells. (**C**) YFP-MS2 (panels **a**–**c**), YFP-GCN5 (panels **d**–**f**), and YFP-Brd2 (panels **g**–**i**) are enriched at the transcription site, marked by Cherry-tTetR-p53-ER. Scale bar represents 5 µm. Scale bar in enlarged inset represents 1 µm. (**D**) Analysis of the percentage of cells with the activators and YFP-MS2 enriched at the transcription site 15 min, 30 min, and 3 hr after activation. 100 cells were analyzed from 3 independent experiments; SEMs (in the form of error bars) are presented in the graphs. (**E**) Analysis of the percentage of cells with accumulations of YFP-GCN5 and YFP-Brd2 at the transcription site 3 hr after activation. 100 cells were analyzed from 3 independent experiments; SEMs (in the form of error bars) are presented in the graphs. (**F**) Measurement of the pixel area of the transcription site in the inactive state (marked by Cherry-lac repressor) and after 3 hrs activation by the VP16 activator (Cherry-tTA-ER with and without α-amanitin pre-treatment) and the p53 activator (Cherry-TetR-p53-ER). Inactive and VP16-activated transcription site data is the same as in Fig. 1D. Area values are averages of 10 cells. SEM (in the form of error bars) and *p* values are presented in the graph. (**G**) Frequency histogram showing the distribution of the blue pixel intensity levels (blue bars) as a measure of the CFP-SKL protein in cells activated for 3 hrs by the VP16 and p53 activators. Black bars represent the background signal. The x-axis is the average fluorescence pixel intensity in each bin on a scale from 0 to 1 and divided into bin sizes of 0.02; the y-axis is the number of pixels in each bin, on a logarithmic scale. The bar beneath the histogram shows the intensity range. Measurements are from 5 independent experiments. Data for the inactive and VP16-activated cells is from the graph in Fig. 1E.

To distinguish between these two possibilities, we compared cells expressing Cherry-tTA-ER (VP16 activator) to cells expressing Cherry-TetR-p53-ER, a construct in which the VP16 acidic activation domain was replaced by the first 70 amino acids of p53, the p53 transactivation domain (p53TAD)(Fig. 9A) [47]. Western blotting of flag-tagged versions of these constructs showed that they are comparably expressed (Fig. 9B). Similar to the VP16 activator, the p53 activator induced the accumulation of YFP-MS2 at the transcription site (Fig. 9C, panels a–c) as well as the recruitment of GCN5 and Brd2 (Fig. 9C, panels d–i).

In cells activated for 3 hours, Cherry-tTA-ER and YFP-MS2 accumulated at the transcription site in 87.6±3.4% and 84.7±4.2% of cells, respectively (Fig. 9D). In contrast, Cherry-TetR-p53-ER and the RNA were detected at the site in only 32.9±3.0 and 36.2±6.3% of cells, respectively (Fig. 9D). Similar results were seen when GCN5 and Brd2 recruitment were evaluated (Fig. 9E). These results, therefore, suggest that activator strength, not binding site number, regulates the percentage of cells in which a gene is activated.

Interestingly, the number of cells activated by VP16 was approximately the same at both the 30 min and 3 hour time points suggesting that the decision to activate is made early and that the number does not increase over time (Fig. 9D). The lower rate of p53 activator induced accumulation is likely due to its lower ability to access the TRE binding sites in the condensed inactive transcription site. Although the p53 activator cannot be detected at the site 15 min after activation, accumulations of YFP-MS2 are detected in 12.7±2.2% of cells indicating that it is a more sensitive detector than the activator.

Additionally, the VP16 activator induced a significantly higher degree of chromatin decondensation than the p53-activator, as determined by measuring the pixel area of the transcription site (Fig. 9C, panels a, d and g, and 9F). The area occupied by the p53-activator bound transcription site was ∼1.4 times larger than the inactive site bound by Cherry-lac repressor, but significantly smaller than the VP16-activated site (Fig. 9F), which is ∼6 times the size of the inactive site. CFP-SKL levels in p53-activated cells were also lower than in VP16-activated cells demonstrating that activator strength also affects the quantity of protein synthesized (Fig. 9G). Taken together, these results indicate that the extent of activation and the ability to overcome a condensed chromatin configuration is due largely to activator strength and not to the number of binding sites upstream of the promoter.

### Transcription is essential for chromatin decondensation

**Figure 10:**
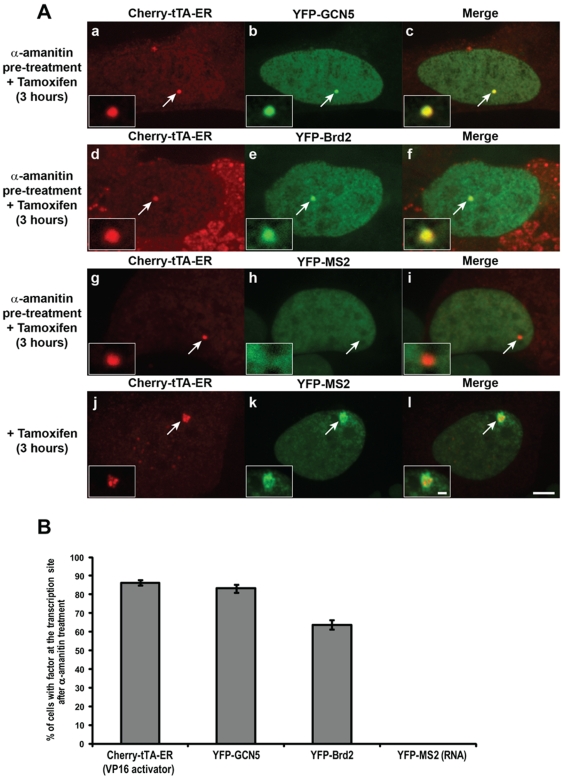
Analysis of the effects of α-amanitin on regulatory factor recruitment. Cells transfected with the indicated factors were pre-treated with α-amanitin for 2 hrs then incubated with tamoxifen for 3 hrs. (**A**) YFP-GCN5 (panels **a**–**c**) and YFP-Brd2 (panels **d**–**f**) were enriched at the transcription site marked by Cherry-tTA-ER. YFP-MS2 was not enriched at the transcription site after α-amanitin treatment (panels **g**–**i**) but did accumulate at the transcription site in untreated cells (panels **j**–**l**). Scale bar represents 5 µm. Scale bar in enlarged inset represents 1 µm. (**B**) Graph shows the percentage of cells with accumulations of each factor at the transcription site after α-amanitin pre-treatment followed by a 3 hr incubation with tamoxifen. 100 cells were analyzed from 3 independent experiments. SEM (in the form of error bars) are presented in the graphs.

To evaluate the role of transcription in chromatin decondensation and regulatory factor recruitment, we pre-treated cells expressing the VP16-activator and YFP-tagged regulatory factors with α-amanitin, added tamoxifen to the media, and evaluated factor recruitment to the transcription site 3 hr later. The lack of YFP-MS2 enrichment confirmed that transcription was inhibited (Fig. 10A, panels g–i, and 10B; Table S1). Interestingly, Cherry-tTA-ER, YFP-GCN5 and YFP-Brd2 were still recruited (Fig. 10A, panels a–f, and 10B). The recruitment of Brd2, which requires it BDs for association (Fig 8A, panels g–i), indicates that the chromatin is acetylated (Fig. 10A, panels d–f). Interestingly, the transcription site in the α-amanitin pre-treated cells did not decondense to the same extent as in untreated cells (∼1.6 times the area of the inactive site) (Fig 9F). This indicates that, although VP16 can still bind to the site and recruit transcriptional regulatory factors, transcription is required to induce large-scale chromatin decondensation.

## Discussion

In this study, we evaluated the recruitment of transcriptional activation factors to a transcription site composed of a multi-copy array of an inducible reporter construct, which allows DNA, RNA and protein to be simultaneously visualized in single living cells. Transcription is initiated when a tetracycline-regulated activator binds to TRE repeats upstream of the CMV minimal promoter in the transgene. In order to track factor recruitment in relation to the activator, we fused tTA (pTet-Off) to both Cherry and the ER hormone binding domain [43] to make the tamoxifen-inducible VP16 activator, Cherry-tTA-ER. The addition of Cherry allows the construct to be used to visualize the active transcription site. The ER domain retains the activator in the cytoplasm until tamoxifen triggers its entry into the nucleus [43]. This cytoplasmic retention serves to both reduce transcriptional leakiness and provides a distinct start point from which to evaluate activation.

Cherry-tTA-ER accumulates at the site ∼7 min after activation. This reflects the time required for it to enter the nucleus, diffuse through the nucleoplasm, and gain access to its binding sites. It is also consistent with previous reports that the glucocorticoid receptor, which also resides in the cytoplasm prior to activation, is detected at the MMTV array within 10 minutes of hormone addition [36]. Heat shock factor (HSF), which is in the nucleus before activation, is highly enriched at the *hsp 70* loci 5 minutes after heat shock [34].

Previously, we showed that this transcription site is heterochromatic in the inactive state [39]. Consistent with those results, we show here by IF and ChIP that histone H3 K9- and H4 K5- and K12- acetylation are not enriched at the inactive site but that they become specifically enriched at the promoter after activation. Therefore, although this transcription site is composed of a repetitive array of a transgene, the sequence elements within it are regulated as they are in single-copy genes.

To investigate the timing of the events regulating transcriptional activation, we tracked the recruitment of GCN5, RNA pol II, Brd4 and Brd2 in relation to the VP16 activator, Cherry-tTA-ER. We wanted to determine whether these factors are recruited in a specific order and how their recruitment correlates with the initiation of RNA synthesis. GCN5, RNA pol II and Brd4 accumulated coincidently with the activator. Interestingly, Brd2 lagged behind Cherry-tTA-ER by ∼2 min. Since Brd2 with mutated BDs does not associate with the site, the timing of its recruitment provides a measure of the time required for the site to become hyper-acetylated. RNA levels also increase simultaneously with the activator suggesting that when activated by the VP16 activator, transcription can proceed before the site is hyper-acetylated.

In general, this assay utilizes transient expression of auto-fluorescently tagged regulatory factors to analyze transcriptional dynamics, although in this study, YFP-RNA pol II and YFP-MS2 were stably expressed. Factor over expression can sometimes introduce effects into a system that can skew results. However, in this study, only YFP-Brd2 detectably increased the rate of activator accumulation at the transcription site (Table S2). This suggests that increased Brd2 expression changes global chromatin structure in such a way that the activator can more rapidly access the TRE binding sites. Yet despite the faster rate of activator binding, Brd2 recruitment is delayed (Table S2) indicating that it requires additional activator-induced regulatory steps to accumulate. Since the Brd2 double BD mutant is not recruited, this suggests that lysine acetylation is the required event. Interestingly, transient expression of GCN5 and Brd4 did not significantly increase the rate of Cherry-tTA-ER accumulation compared to when it was expressed alone demonstrating that factor over expression does not always affect this system (Table S2). However, when it does, it can reveal interesting new information about regulatory factor dynamics.

Importantly, this analysis also allowed us to identify a difference between Brd2 and Brd4, which likely reflects their differential functions. Although highly homologous in their BDs, they are less conserved in their other domains and have been found in different biochemical complexes [48]. For example, Brd4, not Brd2, is a component of select Mediator complexes [48] and is in a complex with P-TEFb, which it recruits to transcription sites where it regulates the transition from initiation to elongation by phosphorylating the RNA pol II C-terminal domain (CTD) at serine 2 [44], [45]. The rapid recruitment of Brd4 to the transcription site in this system is consistent with its role in regulating early events in transcriptional activation. Interestingly, using fluorescent recovery after photobleaching (FRAP) assays, Brd4 was found to have increased chromatin binding during telophase when nuclear factors are beginning to re-associate with the chromatin indicating that these factors also function differently when transcription reinitiates after cell division [49].

Overall, our results indicate that the VP16 activator, Cherry-tTA-ER, induces rapid transcriptional activation and that most of the examined factors accumulate simultaneously with it. As most nuclear factors move rapidly throughout the nucleus by diffusion, this suggests a mechanism for how co-accumulation occurs [50]. In contrast, the rapid initiation of RNA synthesis at the site, coincident with VP16 activator accumulation, is surprising given that transcriptional initiation on naked DNA [51], [52] and the establishment of productive transcriptional complexes in living cells are very inefficient [53]. However, experiments performed by Darzacq and colleagues in the very cell line used in this study determined the maximum rate of RNA pol II elongation without pausing to be 4.3 kb min^−1^ [54]. At this rate, the entire 3.3 kb transcription unit could be transcribed in 46 seconds, Therefore, at least two RNA molecules could be produced from one unit within the 1.5 min imaging interval used in this study. If more than one transgene is initially transcribed by multiple polymerases, a significant pool of RNA would rapidly accumulate at the site. Additionally, each RNA has 24 MS2 repeats, which are sufficient for the detection of a single RNA, making it within our detection limits to visualize even a few RNA molecules at these early time points [55].

It is also possible that transcription begins so quickly because VP16 is a very strong activator. Unlike many cellular activators, which initiate transcription through a cascade of regulatory factor recruitment events regulated by the progressive addition of histone PTMs [30], VP16, itself, may serve as a platform for their direct recruitment. For example, VP16 interacts with the SAGA complex, of which GCN5 is a component [56], [57] and it directly recruits P-TEFb [58], [59]. As such, it may be able to bypass expected intermediate regulatory steps, such as chromatin hyper-acetylation, en route to the initiation of RNA synthesis.

Transcriptional activation rates are also strongly influenced by the number of activator binding sites in a reporter. Increasing the number of NF-kB enhancers bypasses the need for histone acetylation and induces the direct recruitment of transcriptional regulatory complexes [4]. As the transcription unit evaluated in this study has 96 activator binding sites, we compared activation dynamics in cells expressing the VP16-activator to a weaker p53-activator. In p53-activated cells, the RNA, the p53 activator, GCN5 and Brd2 accumulate at only ∼35% of the transcription site compared to ∼90% in VP16-activated cells 3 hrs after induction. This indicates that activator strength rather than binding site number has a much stronger effect on the activation process. Although we cannot definitively say whether the activation is slower in p53-expressing cells because the low signal intensity of this construct at the site and the low percentage of cells activated by it prohibit time-lapse imaging, this analysis provides direct insight into the effects of activator strength on RNA synthesis and chromatin organization at the single cell level.

As most investigations of transcriptional activation have relied on techniques such as chromatin immunoprecipitation (ChIP), nuclear run-on and RNase-protection, in which measurements are taken at widely spaced time points and effects are averaged in cell populations, it has been difficult to determine how transcription is regulated in single cells. Our results show that the number of VP16-activated cells is approximately the same at both the 30 minutes and 3 hours time points indicating that the decision to activate is made early. Although the number of activated cells increases between these time points in p53-activated cells, this is most likely due to detection limits at the early time points. This is also suggested by the fact that the RNA, but not the activator, can be detected 15 min after p53 mediated activation demonstrating that it is the more sensitive detector. This sensitivity difference could also explain why the RNA is detected so rapidly after VP16-activator recruitment in the time-course imaging (Fig. 6).

Our evaluation of transcriptional activation factor recruitment and the condensation state of the transcription site in cells pre-treatment with α-amanitin provides further evidence for the essential role of transcription in driving large-scale chromatin decondensation [1], [40], [41]. Although the VP16 activator, GCN5 and Brd2 are still recruited, the chromatin does not significantly decondense. The fact that Brd2, which requires its BDs for association with the site, is recruited confirms that the site is acetylated and demonstrates that the presence of this PTM is not sufficient for the chromatin to decondense.

### Conclusions

Much of what we know about how genes are activated comes from genomic, biochemical and genetic analyses. However, chromatin is a highly dynamic regulator of gene expression. As changes in chromatin architecture occur within minutes of activation, it is difficult to truly understand the nature of these events when measurements are taken at widely spaced time points and by averaging effects in cell populations. Live cell imaging now makes it possible to monitor transcriptional activation in single cells. In this study, we present one of the first delineations of the timing of transcriptional activation events in single mammalian cells at the earliest stages of activation. Future analyses will allow us to place other regulatory events into this temporal context and to further explore how activation events are coordinately regulated.

## Materials and Methods

### DNA plasmids

Monomeric YFP (mYFP) and monomeric Cherry (mCherry) [42] (gift of R. Tsien) C1, C2, and C3 expression vectors, were made by replacing EGFP in the respective pEGFP vectors (Clontech) with mYFP or mCherry (NheI/BsrGI). To make tTA-ER, the tetracycline transcriptional activator (tTA from pTet-Off; Clontech) was cloned in frame with the estrogen receptor hormone binding domain (ER), which is responsive to both β-estrodiol and tamoxifen and contains the Gly400Val mutation [43], in the pBabePuro:hbER vector (BamHI/EcoRI)(gift of A. Capobianco). mCherry-tTA-ER was made by inserting tTA-ER into mCherry-C3 (KpnI/ApaI). Lentiviral expression plasmid, pLU-mYFP-tTA-ER, was constructed by inserting mYFP-tTA-ER (NheI/BamHI) into a modified pLU vector (gift of A. Ivanov) (XbaI/BamHI). To make mCherry-TetR-p53-ER, TetR was PCRed from pTet-Off and cloned into pCMV-mCherry-C3 (XhoI/HindIII). The ER hormone domain was PCRed and cloned into pCMV-mCherry-TetR-C3 (SacII/BamHI) to make pCMV-mCherry-TetR-ER-C3. The p53 TAD (aa 1–70) was PCRed and cloned into pCMV-mCherry-TetR-ER-C3 (EcoRI/KpnI). Flag-mCherry-tTA-ER and Flag-mCherry-TetR-p53-ER were made by PCRing mCherry-tTA-ER and mCherry-TetR-p53-ER and cloning them into p3xFLAG-CMV-10 (NotI/BamHI). pSV2-mCherry-lac repressor was constructed by replacing EYFP in pSV2-EYFP-lac repressor [60] with mCherry. Mouse GCN5 was cloned into pEYFP-C1 (KpnI/ApaI). Mouse PCAF was cloned into EYFP-C2 (HindIII/Apa1). Human p300 (gift of R. Marmorstein) was cloned into mYFP-C3 (XhoI/Hind III). mYFP-Brd2-C1, mYFP-Brd2 BD(1+2)-Y/F-C1, and mYFP-Brd4-C1 (all are mouse) were made by replacing EGFP with mYFP (AgeI/BsrGI) in constructs provided by K. Ozato [17], [18].

### Cell Culture

The U2OS derived cell line, 2-6-3 [39], was cultured in high glucose Glutamax media (Invitrogen) with 10% tetracycline free FBS (BD Biosciences), 1% pen/strep and 100 µg/ml hygromycin B. YFP-RNA pol II and YFP-MS2 [39] were stably expressed in 2-6-3 cells by selecting in 40 µg/ml G418. Transfections were done using FuGENE 6 (Roche) according to the manufacturer's protocol or electroporation as previously described [39]. Cells transfected with Cherry-tTA-ER were activated with 1 µM tamoxifen (Sigma). For α-amanitin analysis, the 2-6-3 and 2-6-3 YFP-MS2 stable cell lines [39] were transfected overnight with the activator and/or other factors, pre-treated with 100 µg/ml α-amanitin for 2 hrs, and fixed after a 3 hr incubation with tamoxifen.

### Immunofluorescence

For IF, 2-6-3 cells were transfected overnight with SV2-Cherry-lac repressor or Cherry-tTA-ER. Cells were first pre-extracted with 0.5% Triton X-100 in CSK buffer (10 mM PIPES, pH 7.0, 100 mM NaCl, 300 mM sucrose, 3 mM MgCl_2_ with freshly added protease inhibitors) for 5–10 min and then fixed in 3% formaldehyde in 1× PBS for 15 min, both steps at room temperature (RT). Cells were blocked with 3% BSA in 1× PBS for 1 hour at RT and incubated with 1° Abs diluted in blocking buffer. The following Abs were used: RNA pol II 4H8 (Covance, 1∶500), FACTp140 (BD, 1∶100), histone H4 acetyl-K12 (Abcam, 1∶100), histone H4 acetyl-K5 (Upstate, 1∶2000), histone H3 acetyl-K9 (Abcam, 1∶200) and Brd4CA [61] (gift of J. You; 1∶80,000). 2° antibodies were Alexa Fluor AF488 conjugated (Invitrogen) and diluted 1∶3000 in 1× PBS.

### Imaging

For time-lapse imaging, 2-6-3 cells were plated on 40 mm coverslips (Bioptechs Inc., Butler, PA) and transfected overnight with Cherry-tTA-ER alone or co-transfected with YFP-tagged factors; Cherry-tTA-ER was transfected overnight into 2-6-3 cells stably expressing YFP-RNA pol II and YFP-MS2. Coverslips were placed in an FCS2 live-cell chamber (Bioptechs Inc., Butler, PA) with Leibovitz's L-15 medium (Invitrogen) and activation was induced by prefusing ∼3 ml of medium containing 1 µM tamoxifen into the chamber. The chamber and objective lens were maintained at 37°C using heating units (Bioptechs Inc., Butler, PA). The microscope was enclosed in the Incubator BL connected to heating and temperature control units (PeCon GmbH, Erbach, Germany) maintained at 37°C. Images were acquired using a Leica DMI 6000 B inverted automated microscope with HCX PL APO 100x/1.40−0.70 oil objective lens using a 457/488/514nm 30mW Argon-Ion laser for YFP, a 561nm/25mW diode laser for mCherry and a 442nm/70mW diode laser for CFP imaging. To avoid cellular toxicity, the laser powers were decreased to 25% for YFP imaging and 20% for mCherry imaging. In time-course experiments, images were captured using a Hamamatsu electron multiplier CCD digital camera with a 512×512 chip. A Marzhauser point-visiting stage and a Z-drive controlled by COMPIX SimplePCI software were used to collect images at multiple X-Y points. A Yokogawa CSU-10 real-time spinning disk confocal attachment with Nipkow and microlens disks was used to collect stacks of 6 images (0.5 µm increments) every 1 or 1.5 min. Exposure time and gain settings were maintained at specific values for each factor studied (for mCherry tagged factors, 0.3 sec exposure time/gain 200; for YFP-GCN5 0.3 sec exposure time/gain 150; for all other YFP-tagged factors 0.3 sec exposure time/gain 200). Cells with intensity levels registering in the middle of the dynamic range of the camera were selected for imaging in order to ensure that the cells to be analyzed expressed equivalent levels of each factor. For every factor, time course imaging was done on multiple days and on multiple coverslips with 3 cells typically tracked on each coverslip. Cells which went our of focus during imaging, developed a saturated signal at the transcription site, bleached, or showed any kind of aberrant physiological features were not included in the computational analysis. On average, 25–30 cells were imagined for a given factor and ∼10 could be included in the final analysis. Graphs were obtained according to the procedure described below only for cells showing a clear signal to noise ratio of factor accumulation at the transcription site.

For fixed images, cells were plated on 1.5 coverslips, transfected overnight, activated as indicated, and fixed for 15 min in 3% PFA in 1× PBS. Coverslips were mounted in antifade fluorescence mounting medium (1 mg/ml p-phenylenediamine, 90% glycerol in PBS, pH 8.0–9.0 adjusted with Sodium Carbonate/Bicarbonate Buffer, pH 9.2) [39]. Image stacks were taken (0.4 µm increments) using Hamamatsu ORCA-AG camera (1×1 binning; 1344×1024 pixels). Maximum projections were obtained using COMPIX SimplePCI software. Image contrast adjustments were performed using COMPIX SimplePCI and Adobe Photoshop software.

### Image Analysis

Image analysis was done using custom software written in Matlab (The Mathworks). In each time-lapse series, the transcription site was manually selected in the frame when the accumulation of the activator could first be clearly detected (∼10 min after tamoxifen addition). The intensity (arbitrary units) at a point inside the transcription site was compared to the intensity of an outside but nearby point and a threshold, calculated from the intensity difference, was used to define the transcription site boundary. At each time point, activator and factor levels were determined by summing the intensities of all pixels within this boundary and subtracting the background intensity levels. The values in images acquired at earlier time points (before ∼10 min) was determined by applying this first defined boundary to the site. The site was tracked and its location, size, and intensity values were recalculated in all later images. For analysis of YFP-RNA pol II expressing cells, a gamma correction of 2 was applied to each image in both the red and green series. Error bars on the graphed data sets represent one standard deviation across at least three sets of normalized data. The intensity data were fit by performing a nonlinear least-squares regression using the Levenberg-Marquart algorithm in MATLAB with the “lsqcurvefit” function to a logistic model:

Intensity = (A1–A2)/(1+(x/x0)∧p) + A2,
A1 = initial intensity
A2 = final intensity
x0 = the center (intensity value halfway between A1 and A2)
p = power

The timing of the initial accumulations of factors and the activator at the transcription site were determined from the logistic fit. The accumulation start point was defined as the time at which the intensity increased 5% of the difference between the initial and final values. The error estimates of the initial accumulation represent a 90% confidence interval on the estimates of the four model parameters using the function nlparci in MATLAB.

To determine the area of the locus, a point inside and a point outside but nearby the transcription site was manually selected. A threshold calculated from the intensity (in arbitrary units) difference between the two points was used to define the boundary of the transcription site. The “imreconstruct” function in Matlab was used to obtain the region of the transcription site with pixels above the threshold value. The number of pixels identified in the region was then summed for the total locus area of the transcription site.

Quantitative colocalization analysis of fixed images was performed using SimplePCI software. The transcription site was selected manually and Pearson's correlation coefficient (*R_r_*) values with background correction for region of interest (ROI) were calculated. Values from 0.5–1.0 indicate strong colocalization; values from −1.0−0.5 indicate a lack of co-localization. 2D intensity profiles across the transcription site were also obtained for some images.

### Chromatin Immunoprecipitation

For ChIP experiments, cells were infected with the lentiviral expression plasmid, pLU-YFP-tTA-ER for 24 hrs then activated (1 µM tamoxifen) for 3 hrs. ChIP assays were performed using the standard Upstate protocol. The following ChIP grade rabbit polyclonal Abs were used: H4 acetyl-K12 (Abcam), H3 acetyl-K9 (Abcam), histone H4acetyl-K5 (Upstate) and normal rabbit IgG (Abcam). DNA samples from input (In) and antibody-bound (IP) were purified using QIAquick PCR purification kit (Qiagen) and analyzed by real-time PCR using the TaqMan Fast Universal PCR Master Mix (Applied Biosystems) and Applied Biosystems 7500 Fast Real-Time PCR System. Primers and TaqMan probes were designed using Applied Biosystems Primer Express 3.0 software (sequences available upon request). Individual PCR reactions were carried out in triplicates and experiments were repeated three times. Data quantification was performed by applying the modified comparative C_t_ method [62]. Values of % Input were calculated using the following formula IP/In = 2^−dCt^ = 2^−(Ct(IP)−Ct(In))^.

## Supporting Information

Figure S1 Association of the histone acetyl-transferases with the inactive and active transcription site and expression levels of expressed constructs. (A) YFP-GCN5 (panels a–c), YFP-PCAF (panels d–f), and YFP-p300 (panels g–i), are not enriched at the inactive transcription site marked by Cherry-lac repressor. Scale bar represents 5 µm. Scale bar in the enlarged inset represents 1 µm. (B) Western blot showing the levels of the transiently expressed factors used for time lapse imaging: YFP-Brd4, YFP-Brd2 and YFP-GCN5. The lower level of Brd4 compared to Brd2 a result of the less efficient transfer of this higher molecular weight protein.
(0.70 MB TIF)

Figure S2 Association of YFP-RNA pol II with the inactive and active transcription site. Cells stably expressing YFP-RNA pol II were transfected with Cherry-lac repressor, to mark the inactive transcription site (panels a–c). Cherry-tTA-ER marks the transcription site 3 hrs after activation induced by tamoxifen (panels e–g). Intensity profile shows that YFP-RNA pol II (green line) surrounds but does not co-localize with the inactive site (red line) (panel d). YFP-RNA pol II significantly co-localizes with Cherry-tTA-ER (panel h). Yellow lines in enlarged insets in c and g show the path starting at the asterisk through which the red and green intensities were measured (panels d and h). Scale bar represents 5 µm. Scale bar in the enlarged inset represents 1 µm.
(2.89 MB TIF)

Table S1 Analysis of factor co-localization with the transcription site.
(0.05 MB DOC)

Table S2 Summary of the recruitment time analyses from time series images of activator and regulatory factor accumulation at the transcription site during activation. The gray shaded column is the 5% accumulation threshold, which is marked by arrows in the graphs in the figures.
(0.03 MB DOC)

Movie S1 Cherry-tTA-ER was transiently transfected into 2-6-3 cells and transcription was induced by the addition of tamoxifen. Frames were collected every minute for ∼40 min. Movie display rate is 8 frames per second. Still images from this movie are shown in Figure 2A.
(3.41 MB AVI)

Movie S2 Cherry-tTA-ER and YFP-GNC5 were transiently transfected into 2-6-3 cells and transcription was induced by the addition of tamoxifen. Frames were collected every 1.5 min for ∼40 min. Movie display rate is 8 frames per second.
(0.91 MB AVI)

Movie S3 Cherry-tTA-ER was transiently transfected into a 2-6-3 cell line stably expressing YFP-RNA pol II. Transcription was induced by the addition of tamoxifen. Frames were collected every 1.5 min for ∼40 min. Movie display rate is 8 frames per second.
(1.20 MB AVI)

Movie S4 Cherry-tTA-ER was transiently transfected into a 2-6-3 cell line stably expressing YFP-MS2. Transcription was induced by the addition of tamoxifen. Frames were collected every 1.5 min for ∼40 min. Movie display rate is 8 frames per second. Still images from this movie are shown in Figure 6.
(1.04 MB AVI)

Movie S5 Cherry-tTA-ER and YFP-Brd4 were transiently transfected into 2-6-3 cells and transcription was induced by the addition of tamoxifen. Frames were collected every 1.5 min for ∼40 min. Movie display rate is 8 frames per second.
(13.33 MB AVI)

Movie S6 Cherry-tTA-ER and YFP-Brd2 were transiently transfected into 2-6-3 cells and transcription was induced by the addition of tamoxifen. Frames were collected every 1.5 min for ∼40 min. Movie display rate is 8 frames per second.
(0.75 MB AVI)
