# Supplementary figures and images for: Single Cell Analysis of Transcriptional Activation Dynamics
Source: PLoS One. 2010 Apr 21;5(4):e10272. doi: 10.1371/journal.pone.0010272 (PMC2858074; doi:10.1371/journal.pone.0010272)

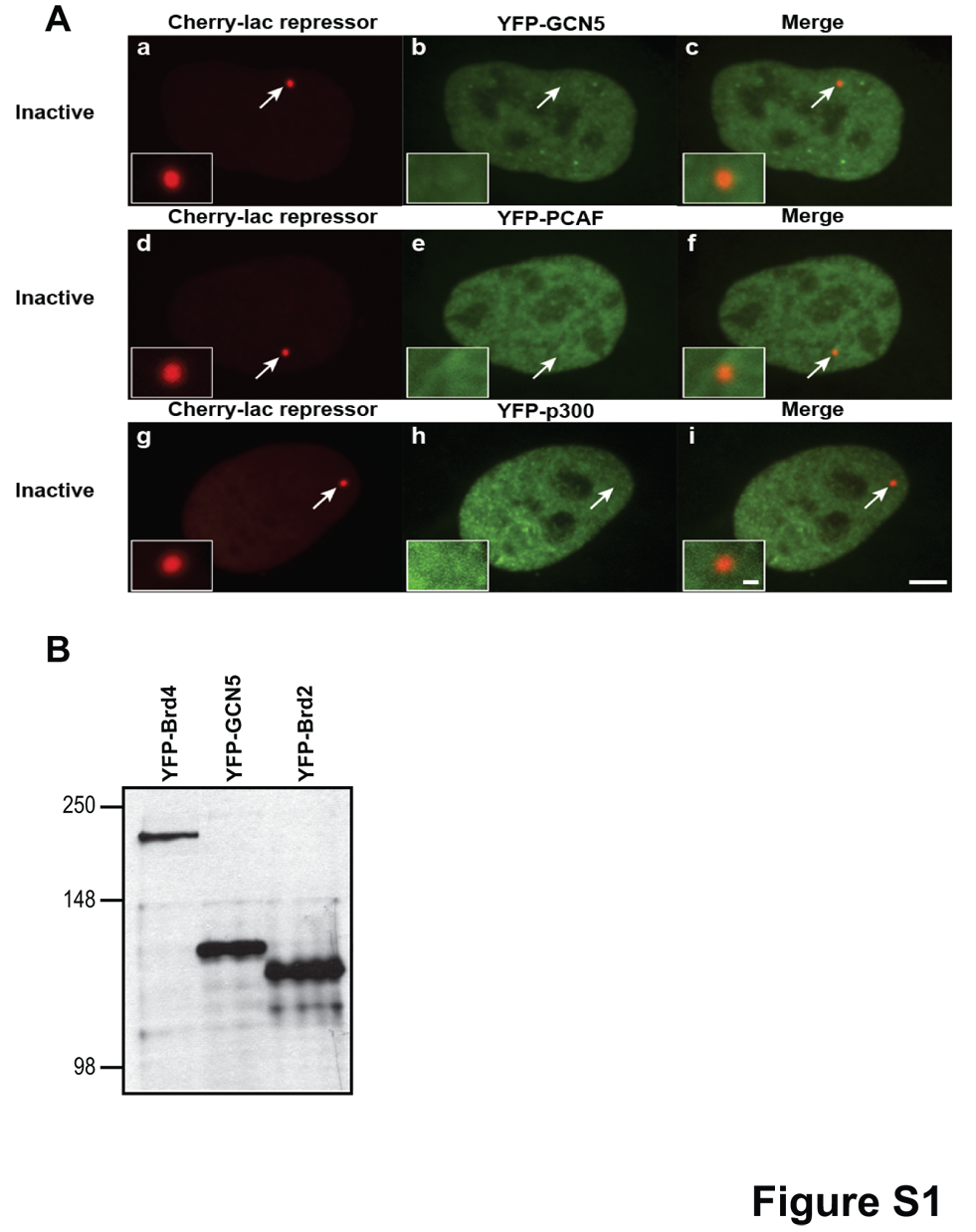

Supplement: Figure S1 — Association of the histone acetyl-transferases with the inactive and active transcription site and expression levels of expressed constructs. (A) YFP-GCN5 (panels a–c), YFP-PCAF (panels d–f), and YFP-p300 (panels g–i), are not enriched at the inactive transcription site marked by Cherry-lac repressor. Scale bar represents 5 µm. Scale bar in the enlarged inset represents 1 µm. (B) Western blot showing the levels of the transiently expressed factors used for time lapse imaging: YFP-Brd4, YFP-Brd2 and YFP-GCN5. The lower level of Brd4 compared to Brd2 a result of the less efficient transfer of this higher molecular weight protein. (0.70 MB TIF) [file pone.0010272.s001.tif]

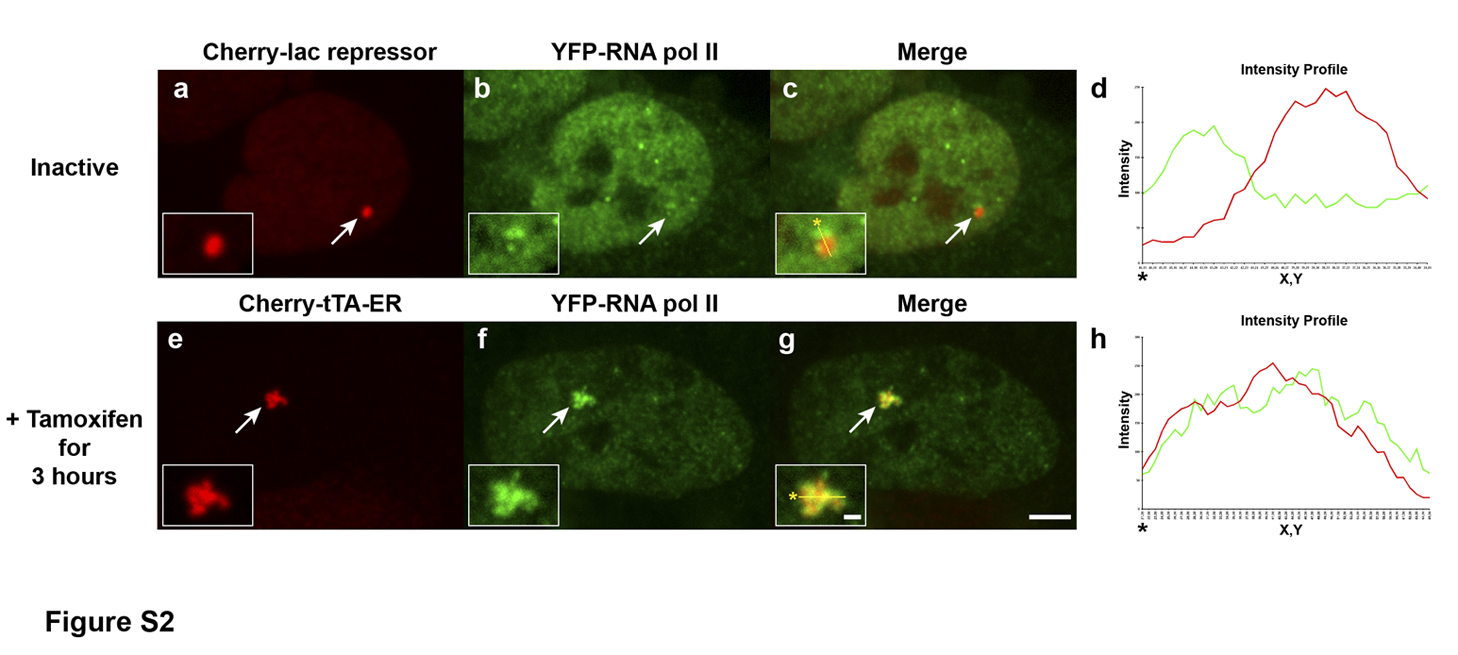

Supplement: Figure S2 — Association of YFP-RNA pol II with the inactive and active transcription site. Cells stably expressing YFP-RNA pol II were transfected with Cherry-lac repressor, to mark the inactive transcription site (panels a–c). Cherry-tTA-ER marks the transcription site 3 hrs after activation induced by tamoxifen (panels e–g). Intensity profile shows that YFP-RNA pol II (green line) surrounds but does not co-localize with the inactive site (red line) (panel d). YFP-RNA pol II significantly co-localizes with Cherry-tTA-ER (panel h). Yellow lines in enlarged insets in c and g show the path starting at the asterisk through which the red and green intensities were measured (panels d and h). Scale bar represents 5 µm. Scale bar in the enlarged inset represents 1 µm. (2.89 MB TIF) [file pone.0010272.s002.tif]
