# Supplementary material for: Single Cell Analysis of Transcriptional Activation Dynamics
Source: PLoS One. 2010 Apr 21;5(4):e10272. doi: 10.1371/journal.pone.0010272 (PMC2858074; doi:10.1371/journal.pone.0010272)
Supplement: Table S1 — Analysis of factor co-localization with the transcription site. (0.05 MB DOC) [file pone.0010272.s003.doc]

| **Image** | **Pearson's Correlation Coefficient (*R_r_*) for selected ROI^a^** |
| --- | --- |
| tTA-ER + LacI OFF | 0.0823 |
| tTA-ER + LacI ON | 0.8498 |
| tTA-ER + MS2 ON | 0.3817 |
| H4 AcK12 + LacI OFF | -0.0145 |
| H4 AcK12 + tTA-ER ON | 0.9008 |
| H4 AcK5 + LacI OFF | -0.2329 |
| H4 AcK5 + tTA-ER ON | 0.5807 |
| H3 AcK9 + LacI OFF | 0.1099 |
| H3 AcK9 + tTA-ER ON | 0.7433 |
| GCN5 + LacI OFF | 0.0258 |
| GCN5 + tTA-ER ON | 0.9316 |
| PCAF + LacI OFF | 0.1042 |
| PCAF + tTA-ER ON | 0.8982 |
| p300 + LacI OFF | -0.1044 |
| p300 + tTA-ER ON | 0.7172 |
| RNA pol II + LacI OFF | -0.2429 |
| RNA pol II + tTA-ER ON | 0.6300 |
| FACTp140 + LacI OFF | -0.2170 |
| FACTp140 + tTA-ER ON | 0.6509 |
| Brd4 + LacI OFF | -0.1475 |
| Brd4 + tTA-ER ON | 0.8472 |
| end Brd4 + tTA-ER ON | 0.7717 |
| Brd2 + LacI OFF | -0.4488 |
| Brd2 + tTA-ER ON | 0.7752 |
| Brd2 BD(1+2)Y-F + tTA-ER ON | 0.1563 |
| GCN5 + TetR-p53-ER ON | 0.8532 |
| Brd2 + TetR-p53-ER ON | 0.7240 |
| GCN5 + tTA-ER ON after amanitin | 0.8832 |
| Brd2 + tTA-ER ON after amanitin | 0.8996 |
| tTA-ER + MS2 ON after amanitin | -0.0994 |

**^a^** Values from 0.5 to 1.0 indicate strong colocalization, whereas values from -1.0 to 0.5 indicate absence of colocalization.
