# Supplementary material for: Single Cell Analysis of Transcriptional Activation Dynamics
Source: PLoS One. 2010 Apr 21;5(4):e10272. doi: 10.1371/journal.pone.0010272 (PMC2858074; doi:10.1371/journal.pone.0010272)
Supplement: Table S2 — Summary of the recruitment time analyses from time series images of activator and regulatory factor accumulation at the transcription site during activation. The gray shaded column is the 5% accumulation threshold, which is marked by arrows in the graphs in the figures. (0.03 MB DOC) [file pone.0010272.s004.doc]

| **Movie** | **Times (min) at x% of total accumulation** | | | **Time difference between activator and factor accum.** |
| --- | --- | --- | --- | --- |
|  | **x=2.5** | **x=5** | **x=10** |  |
| VP16 Activator | 6.0 ± 0.9 | 7.5 ± 1.0 | 8.8 ± 1.1 | n/a |
| VP16 Activator  GCN5 | 5.6 ± 0.9  5.6 ± 1.0 | 6.9 ± 1.0  7.0 ± 1.1 | 8.7 ± 0.9  8.7 ± 1.1 | 0 |
| VP16 Activator  RNA pol II | 4.7 ± 1.5  5.0 ± 1.8 | 6.3 ± 1.8  6.5 ± 2.1 | 8.5 ± 2.0  8.4 ± 2.4 | 0 |
| VP16 Activator  MS2 | 5.2 ± 1.1  5.3 ± 1.2 | 6.6 ± 1.1  6.7 ± 1.3 | 8.3 ± 1.2  8.6 ± 1.4 | 0 |
| VP16 Activator  Brd4 | 5.6 ± 0.9  5.9 ± 0.8 | 6.9 ± 1.0  7.0 ± 0.9 | 8.8 ± 1.0  8.4 ± 0.8 | 0 |
| VP16 Activator  Brd2 | 3.8 ± 1.0  6.1 ± 1.1 | 4.9 ± 1.1  7.0 ± 1.1 | 6.5 ± 1.3  8.2 ± 1.1 | ~2 min |
